# Long-term follow-up of newborns at neurological risk

**DOI:** 10.1186/s13052-019-0629-7

**Published:** 2019-03-18

**Authors:** Enrico Gasparrini, Francesca Rosati, Maria Teresa Gaetti

**Affiliations:** 1 Department of Pediatrics and Neonatology - Hospital of Macerata, Macerata, Italy; 2 Autonomy and Life Rehabilitation Center, Falconara, Italy; 3 Department of Pediatrics and Neonatology - Hospital of Jesi, Jesi, Italy

**Keywords:** Quality of life, Newborns at neurological risk, Follow-up, Neurological outcome, Neuro-evolutionary development

## Abstract

**Background:**

In order to give a new contribution to the knowledge of the psycho-physical, behavioral and socio-relational development of the individuals who were born at neurological risk, we have carried out a research work through a retrospective and observational analysis in such people, followed in their neuro-evolutionary development from the Department of Pediatrics and Neonatology of the Hospital of Jesi.

The purpose of this work is to value the quality of life of the individuals born at neurological risk at a distance of time from the birth.

In the literature only recently there are studies on the quality of life of some categories of people, but survey does not seem to be performed in individuals previously born at neurological risk.

**Methods:**

A statistical descriptive and inferential survey has been carried out on 812 individuals who were born at neurological risk, 442 preterm newborns and 370 term newborns, followed from 1977 until to 2007. They were classed in order to their age at the time of our observation. We have submitted the entire sample to a Questionnaire to investigate some areas of their life, ranging from their clinical and psycho-social history to their personal coming of life. Then the same persons, subdivided according to the various age groups, were subjected to other Questionnaires on the quality of life, internationally used.

**Results:**

Neurological outcomes were found in 14.7% of the preterm newborns and in 6% of the term newborns, with a significant correlation between neurological outcomes and gestational age, low birth-weight, hypoxic-ischemic encephalopathy and low APGAR-index. Neuro-disabilities were found prevalently belong to the small for gestational age preterm newborns. A low quality of life emerged in those who had neurological outcomes.

**Conclusions:**

Our study on the individuals who were born at neurological risk, analyzed at a distance, shows that a good health is associated with a good quality of life, while a low quality of life occurs to those who had neurological outcome, especially in the physical, cognitive, emotional and socio-relational aspects.

As far as the few neurological outcomes which we have found in this survey, we think that they are due, other than to the natural factors, also to the high quality of the obstetric and neonatal care, to the early habilitation physiotherapy and to the important collaboration with the family.

## Background

The progress achieved in the last few decades in the preventive, diagnostic and therapeutic fields regarding the assistance to pregnant women and the care of the newborns have led to a significant drop in the mortality rate of the newborns at neurological risk. However, despite the progress in the medical field has allowed to increase the likelihood of survival even of preterm newborns with low gestational age (< 32 weeks) and/or very low birth-weight (< 1500 g, called VLBW, and <  1000 g, called ELBW), in recent years there has been no reduction in the incidence of developmental disorders of various kinds in these children [1, 2].

So if on the one hand, thanks to recent technological and pharmacological discoveries in the prenatal, perinatal and neonatal care, there has been a significant reduction of the risk for mortality and permanent diseases, on the other hand many studies have documented that these persons, compared to those borns at-term, are more at risk for the onset of disorders of neuro-psychological development, which tend to increase with the decrease of the gestational age, such as, for example, postural and motor deficits, dyspraxies, behavioral changes, disorders of language, attention deficit and hyperactivity, relational difficulties, affective disorders, learning difficulties, impairment of visuo-motor integration and quality of life [3].

These deficits often emerge in preschool and school age, proving how the preterm birth has consequences also on the children who do not present clear clinical manifestations that can be diagnosed early.

In the light of these observations, more attention has been put to the medium and long term effects of the preterm birth, not only on the level of physical and/or cognitive functioning, but also on that of psychosocial and emotional adaptation. In particular, it seems reasonable to argue that one of the most relevant areas in the field of the neonatology, the neuropsychiatry and the developmental psychology is to optimize the social and environmental adaptation and the quality of life related to the health status of the preterm baby.

In Italy, in 2017, 458,151 children were born [4]: about 10% of them were born preterm (before the 37th week of gestation) and 1% were born before the 32th week of gestation [5].

Of course the neurological risk is not due only to the preterm birth, but it may be due to family-genetic factors or to alterations during the pregnancy, during or after the delivery.

In the recent years the introduction of new instrumental techniques for the morphological, functional and metabolic study of the brain since the neonatal age has provided an important contribution to the diagnosis and the prognosis of the neurological damage.

Although it is certainly important to establish the connections between the neuroimaging (ultrasound, computed tomography, cerebral magnetic resonance imaging), the evoked potentials (visual, auditory, neurosensory), the electroencephalography and the type of the brain damage, its extension and location, its onset, the type and severity of the neurological deficit, it is also true that this assessment does not always give satisfactory results, especially with regard to the minor brain injuries (transient hyperechogenicity, microcystic periventricular leukomalacia, intraventricular not extensive hemorrhages).

The neuroradiological and the electroencephalographic findings do not always correlate with the severity of the psychomotor outcome. In fact, cases with normal or mildly compromised psychomotor development have been described in the presence of extensive cystic leukomalacies and, conversely, cases of children in whom the presence of no-cystic leukomalacia has led to the development of cerebral palsy.

It is therefore evident that the alone neurological instrumental semeiology does not allow to define the categories of newborns at neurological risk. Therefore it is necessary that it is always flanked by a valid neurological clinical semeiology that, used before the development of the technologies of recent years, has always had its validity [6] and, nowadays, is further enriched thanks to the greater knowledges, especially in the neuro-functional field [7–11].

Near to the relief of the cerebral lesion, which should be documented in as much detail as possible, the integrity of the central nervous system and the ability to compensate for the possible damage must be evaluated (the brain plasticity has exceptional potentialities in the first months of the life). The clinical evaluation must ascertain the presence and the degree of the brain dysfunction regardless of whether or not macroscopically documented brain lesions are found.

The scientific studies of the neurological clinical semeiology of the newborn began in the years 1950–1960 with Andrè Thomas, Saint Anne Dargassies, Joppich and Schulte, although various “neonatal reflexes” had already been reported by Magnus and Klein in 1912 and Moro had gone beyond the neurophysiological-phylogenetic meaning assigned initially to them and had suggested their use for neurological diagnostics.

But it is above all in the last fifty years that, for the greater survival of infants of ever lower weight and the consequent need to make them a good quality of life, especially in the neuropsychic field, more and more neurological evaluation techniques have been developed to attribute also a prognostic meaning, as well as a diagnostic one.

In particular, Prechtl, Cioni and Ferrari have developed a method for assessing the neuromotor development of the newborn, based on the observation of the spontaneous motility (“General Movements”). This instrument, that can be applied early also on the delicate newborns because it does not involve the manipulation of the child, has shown a good sensitivity and a good specificity, allowing a reliable predictability and an early diagnosis of pathological development [7–10].

In order to give a contribution to the knowledge of the psycho-physical, behavioral and socio-relational development of the individuals who were born at neurological risk, and, above all, to the knowledge of their quality of life at a distance of birth, induced also by the scarcity of studies on this argument [12–14], we have carried out a retrospective and observational analysis in such people, who had been followed-up for years in their neuro-evolutionary development from the Department of Pediatrics and Neonatology of the Hospital of Jesi.

The purpose of our study was:

- to evaluate the quality of life of the individuals at a distance of time from their birth
- to consider their path of the growth and of the intellectual maturation
- to determine their auxological development
- to analyze the psychosocial and affective support from their family and from the Socio-health Services.

## Methods

We started our research by examining the medical records filed in the Department of Pediatrics and Neonatology of the Hospital of Jesi concerning 1704 patients who were born at neurological risk, excluding those affected by genetic and metabolic diseases, followed-up in the same Department in the period between 1977 and 2007.

These people were 6% of 28,032 individuals born in that period: 854 were born preterm and 850 were born at-term, both born at neurological risk. Most of them (92.8%) were born at the same Hospital (inborn), only some of them (7.2%) came from other Structures (outborn).

The parameters that we took into consideration, concerning them, were the following:

- Personal data (age, gender)
- Gestational age
- Birth weight: AGA (weight between 10th and 90th percentile), SGA (<10th percentile), LGA (> 90th percentile)
- APGAR score at first minute < 7
- Type of delivery (eutocic/distocic)
- Diagnosis at birth
- Outcome in the first year of life
- Abilitive physiotherapy (yes / no).

The aforementioned individuals were invited to take part to the study by a letter, in which they were informed that they would be contacted by one of our Operators for a meeting at the Department of Pediatrics and Neonatology of the Hospital of Jesi.

The choice of “face to face” was based on the possibility to obtain a higher quality of answers, better compliance and a higher percentage of respondents.

We have submitted all the people who responded to our invitation, regardless of age, to a *Questionnaire* to investigate some areas of their life, ranging from their clinical and psycho-social history to their personal coming of life (for example: health state, social position, level of study, social and family relationships, enabling therapy, etc.).

We then subjected the same persons, subdivided according to the various age groups, to other *Questionnaires on the quality of life*, internationally used [15, 16]:

- *The Tap QoL (TNo-AZL Preschool Children Quality of Life Questionnaire;* *1–7 years):* given to the parents of the children who were born at neurological risk. It consists of 43 items that investigate the various psycho-physical areas of the subject examined. The areas concern the sleep, the physiological functions related to the gastroenteric, musculoskeletal and respiratory apparatus, the cognitive, communicative, emotional and social-relational sphere.
- *PedsQoL (Pediatric Quality of Life Inventory,* *8–14 years):* it is a generic questionnaire developed on 23 items that investigate the areas related to the physical, emotional, social and scholastic functions.
- *HU13 (Health Utilities Index Mark3,* *15–17 years):* 45 items that investigate the areas related to the physical, cognitive, emotional and pain-related functions.
- *SF36* and *ID-5D VAS (Euro Quality 5 Dimensions and visuo-analogue,* *18–32 years):* both tests analyze, through a scale from 0 to 100, the perception of the state of the physical and emotional health, investigating some areas of exploration: physical function, physical pain, general perception of own state of health, vitality, social activities, limitations of role due to emotional problems, mental health.
- *TAT proprioceptive test (Thematic apperception test or Murray test,* *15–32 years):* examines the psychic asset from an analytical point of view and evaluates both the possible effects that being born at neurological risk has in the parental relationship at the inter/intrapsychic level (“dialogue with the internal parental images”), both the functional and pathological defensive mechanisms that the person activates in his experiential path. In detail it is a test that uses projection phenomena starting from the tables that are presented to the individual who elaborates a “story”, whose interpretation highlights attitudes, conflicts, defensive mechanisms which are often unconscious. It is composed from 31 tables and includes differentiated series that are suitable for young or adult persons, male or female.

Therefore a descriptive and inferential statistical survey was carried out.

The absolute frequencies and the percentages according to term and preterm newborns were calculated for the categorical variables. The comparison between the two groups was made by the *Chi-square Test (χ*^*2*^*)* and the *Fischer Exact Test*.

The quantitative variables were instead synthesized using the median as a measure of centrality and the first and third quartile as a measure of dispersion. The groups were compared by the *Wilcoxon-Mann-Withney Test.*

## Results

Invited to participate in the study 1704 individuals who were born at neurological risk and followed in the period between 1977 and 2007, 812 individuals (about 50%) came in a year: 442 preterm borns and 370 term borns. These constitute the sample of our survey.

**Fig. 1:**
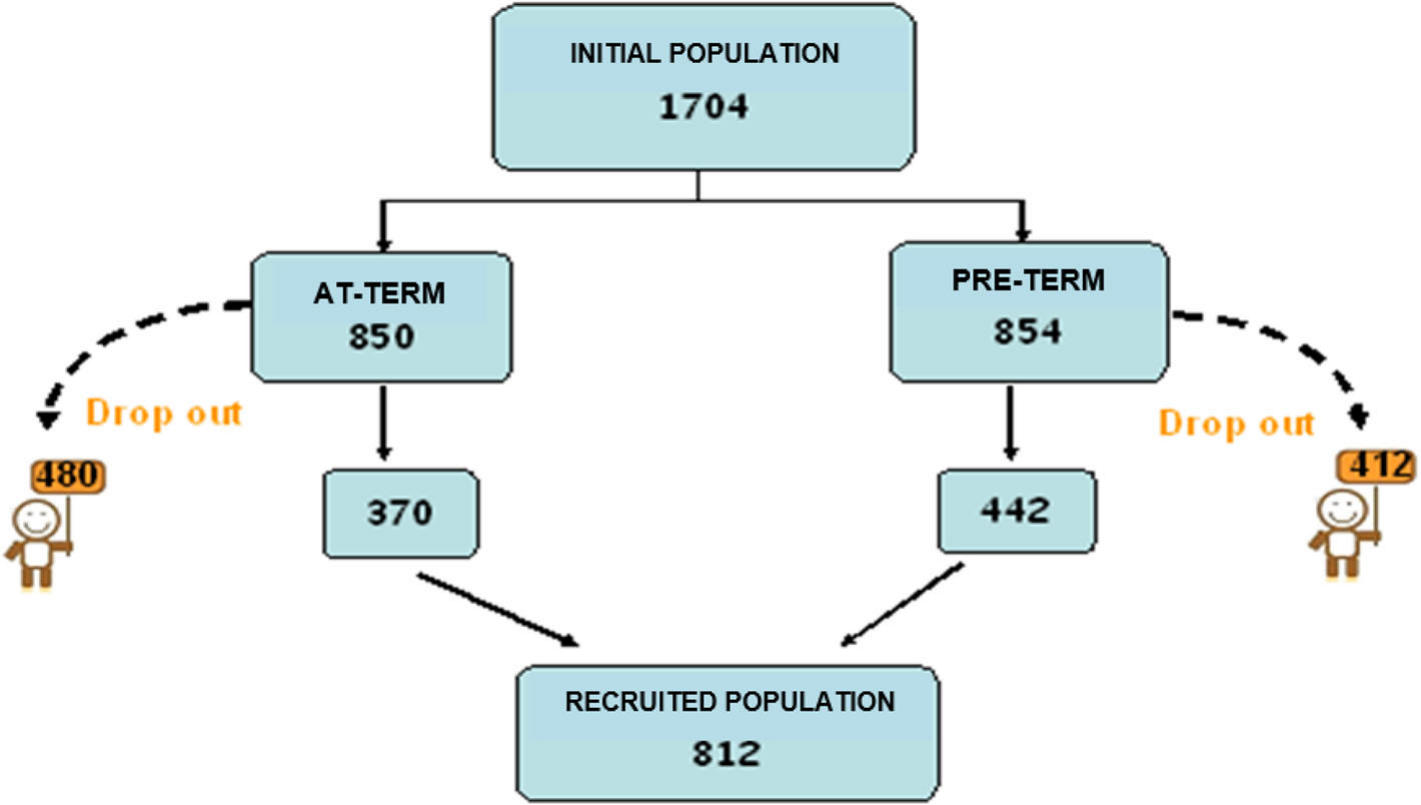
The outline of the individuals invited and recruited to take part to the study

The drop-out (slightly greater for the term borns) was mainly determined by the inability to find personal informations (telephone numbers, addresses, etc.) and by the lack of response from someone. These data are shown in Fig. 1.

The classification based on the degree of prematurity of the individuals examined is reported in Table 1.

**Table 1:** The degree of prematurity of the individuals examined

Level of prematurity	%	Average Gestational Age
*34* ^*+ 0*^ *–36* ^*+ 6*^ *weeks of GA*	72	*35.14 ± 0.86*
*32* ^*+ 0*^ *–33* ^*+ 6*^ *weeks of GA*	21	*33.06 ± 0.45*
*28* ^*+ 0*^ *–31* ^*+ 6*^ *weeks of GA*	6.6	*29.51 ± 1.05*
*< 28* ^*+ 0*^ *weeks of GA*	0.4	*26.67 ± 0.52*

**Table 2:** The prevalence of neurological outcomes

Neurological Outcomes	N° Preterm newborns	(% on 442)	N° term newborns	(% on 370)
Neuro-sensory deficit	16	3.6%	9	2.4%
Neuro-motor deficit	14	3.2%	3	< 1%
Psycho-motor delay	14	3.2%	4	< 1%
Language delay	11	2.5%	2	< 1%
Epilepsy	2	< 1%	2	< 1%
Hydrocephalous	3	< 1%	0	----
Walking deficit	5	< 1%	2	< 1%
*Tota*l	65	14.7%	22	6%

We then subdivided these people, selected on the basis of their age at the time of our observation, in the following group:

- 1–7 years
- 8–14 years
- 15–17 years
- 18–32 years.

From the analysis carried out in the phases of the experimentation on 812 individuals of the sample it results that 14.7% of the preterm newborns and 6% of the term newborns have had pathologic outcomes (Table 2).

The presence of pathologic outcomes is statistically correlated (*p* < 0.001) to the presence of hypoxic-ischemic encephalopathy at the birth, to the APGAR score of less than 7 at the first minute of life, to the neonatal gestational age and at to the birth weight.

Among preterm newborns, the incidence of problems increased as the neonatal gestational age was reduced and, among them, the prognosis of the small for the gestational age (SGA) newborns was worse; on the other hand, the kind of delivery and twin birth have no significance (Table 3).

These results are in accordance with our previous survey carried out on infants of low birth weight at 5 years of age [16] and with the data of the literature.

From the questionnaire submitted to the people who responded to our invitation regardless of the age, it has emerged that were no statistically significant differences between preterm and term newborns as regards the auxological aspect. In fact, there was a normal stature-weight growth in almost all the children examined, also if, however, 6.6% of the SGA newborns had a short stature (< 3° or -2DS) and 20% of them were obese.

Almost all the children followed in the follow-up who, during the first months of life, had presented signs of a predictive neurological pathology (“symptomatic-risk children”) or an established neuro-evolutionary pathology had been subjected to early habilitation physiotherapy (96.8% of preterm newborns and 94.8% of term newborns respectively, p 0.21).

All the individuals have had a psycho-social support from their families, even if in 43% of cases it was not found to be qualitatively valid, because of an altered relationship between the parental figures, resulting from an emotionally perceived pathological experience.

All the patients with more or less severe problems of neuro-evolutionary development had benefited from the Neuro-psycho-pedagogical and social District Services, even if these have not always been adequate and efficient.

There were statistically significant differences between term and preterm newborns with regard to sleep disorders (p 0.01) and school difficulties (0.04).

At last, there were statistically significant differences in the level of employment: in fact, study or work 58.6% of term newborns versus 48.2% of the preterm newborns (*p* < 0.0001).

As for the quality of life, which has been evaluated by comparing it to a scale of values ranging from 0 to 100, where 0 represents the minimum and 100 maximum satisfaction, it has been seen that the group of preterm newborns has presented an average value equal to 50 against a value equal to 69 of those born at term.

In detail, as reported in the Tables 4,5,6,7, among the group of the preterm newborns and the group of the term newborns, the following statistically significant differences emerged in the various age groups:

**Table 3:** The neurological outcomes and associated factors

Neurological Outcomes	*p*
Hypoxic-ischemic encephalopathy	0.001
APGAR score at first minute < 7	0.001
Twin birth	0.15
Gestational age	0.001
Kind of delivery	0.971
Birth weight (SGA, AGA, LGA)	0.001

**Table 4:** TNo-AZL Preschool Children Quality of Life Questionnaire (1-7 years)

Quantitative variables: median (1 and 3 quartile) Wilcoxon test	*Term (n. 70)*	*Preterm (n. 70)*	*p*
physical impact	36 (35–36)	34 (31–36.5)	*0.01*
emotional impact	6 (6–6)	5 (5–6)	*< 0.0001*
social impact	14 (13–14)	14 (12–14)	*0.21*
cognitive impact	8 (8–8)	7 (7–7)	*< 0.0001*
Total score (0–100)	74.4 (73.3–74.4)	69.8 (63.4–75.6)	*0.00*

**Table 5:** Pediatric Quality of Life Inventory (8-14 years)

Quantitative variables: median (1 and 3 quartile) Wilcoxon test	*Term (n. 70)*	*Preterm (n. 71)*	*p*
physical impact	90.6 (71.9–93.8)	84.4 (78.1–87.5)	*0.06*
emotional impact	85 (80–85)	80 (60–90)	*< 0.0001*
social impact	100 (0–100)	75 (10–90)	*< 0.0001*
scholastic impact	85 (10–90)	50 (30–70)	*0.08*
Total score (0–100)	90 (85.2–94.7)	78.3 (71.1–83.9)	*< 0.0001*

**Table 6:** HU13 (Health Utilities Index Mark3) (15-17 years)

Quantitative variables: median (1 and 3 quartile) Wilcoxon test	*Term (n. 47)*	*Preterm (n. 36)*	*p*
impact of auditory, visual, linguistic capacity	1 (0.93–1)	0.85 (0.78–0.93)	*< 0.0001*
impact of motor capacity	1 (1–1)	0.75 (0.5–1)	*< 0.0001*
impact of emotional capacity	1 (1–1)	0.75 (0.5–1)	*< 0.0001*
impact of cognitive capacity	1 (1–1)	1 (0.95–1)	*0.05*
perception of pain	1 (0.75–1)	1 (0.75–1)	*< 0.0001*
Total score (0–1)	0.96 (0.96–1)	0.91 (0.87–0.93)	*< 0.0001*

**Table 7:** Euro Quality 5 Dimensions and visuo-analogue (18-32 years)

Quantitative variables: median (1 and 3 quartile) Wilcoxon test	*Term (n. 183)*	*Preterm (n. 265)*	*p*
impact of motor capacity	1 (1–1)	1 (1–1)	*0.41*
self-care	1 (1–1)	1 (1–1)	*0.41*
pain/discomfort	1 (0.6–1)	1 (0.4–1)	*< 0.0001*
anxiety and depression	1 (0.3–1)	1 (0.6–1)	*< 0.0001*
VAS (0–100)	88 (83–95)	85 (80–96.7)	*0.24*

A low quality of life has emerged in those persons who had neurological outcomes. The most compromised areas were related to the following aspects:

- physical: 27.3%.
- social: 18%.
- cognitive: 14.3%.
- emotional: 14.2%.

## Discussion

From the survey carried out on individuals who were born at neurological risk of our case series it emerges that few neurological outcomes are found. It is to be thought that this is partly due to natural factors, but above all to the quality of the obstetric care and to the timeliness and the appropriateness of the neonatal care, to the early habilitation physiotherapy, to the important collaboration with the family (“the therapeutic alliance”).

The data analyzed show that the satisfactory physical state of the individuals in question (the low percentage of neurological outcomes at a distance, stature-weight growth in the norm in the majority of cases) is associated with an average satisfactory quality of life.

The areas investigated which have negative implications on the quality of life are related to the physical, cognitive, emotional and socio-relational aspects. In fact, a low quality of life emerges, especially in the socio-relational and affective aspects, in those who had neurological outcomes.

Regarding the emotional aspect, that includes the basic emotions (fear, anxiety, agitation, joy), analyzed through various questionnaires, may be say that all these emotions are enclosed in a sort of emotional container whose space and whose form are shaped by the relationship with the mother, who represents the first emotionally charged relationship. While in the normal conditions the newborn can immediately benefit from the maternal containment function, in cases where the baby is prematurely separated from the reference figure (pre-term birth and/or neurological risk at birth, which implies the hospitalization), he is alone to manage his emotional states and to trace the boundaries of his internal world, often in a labile and dysfunctional way.

This problem, as shown by our study, has repercussions on the structuring of personal identity.

Also the post-natal emotional stress of the parents may be associated with the behavioral disturbances and the cognitive development of the child, together with other factors related to familiarity, both of a hereditary nature and of an educational peculiarity [17].

What we have found in the sample is the difficulty to manage the emotions and to face up to the frustrations and anxieties. Since the interpersonal relationships represent the privileged stage of the emersion of the emotional states, it is clear that the problem has repercussions in the social sphere: family, friendships, school, work.

A good contribution to overcome this anguished and suffered experience of the child and of his parents is given by processes of the humanization (care, gentle handling, skin to skin, minimal manipulation, etc.), which from several years are applied in the Departments of Neonatology and Neonatal Intensive Care, especially with the NIDCAP (Newborn Individualized Developmental Care and Assessment Program) [18, 19], method which, beyond the stabilization of the vital functions and of the health services, certainly of priority importance, supports the relational needs of the newborn and of his parents and encourages their affective link, using appropriately the technology resources and reducing the inconveniences and the disadvantages associated with the hospitalization [19, 20].

## Conclusions

From our research on a population of individuals born at neurological risk, it emerges that few neurological outcomes have been found in these subjects and that their quality of life, evaluated at a distance from the birth, is in average discreet, better in term borns.

As it was logical to expect, a low quality of life has been demonstrated, especially in the social-relational and affective aspects, in those who had neurological outcomes.

The good results obtained are undoubtedly to be attributed largely to the quality and timeliness of the obstetric and neonatal care, to the precociousness of habilitation physiotherapy and to the collaboration with the family, as well as to the humanization processes.

Since it has been shown that the humanization is a model of care that takes into account the relational needs of the newborn and of the parents and strengthens their link, the psycho-physical development of the child and increases the effectiveness of the care, we hope that these processes are always improved and applied in every part of the world, along with an increasing focus on the observation of the clinical performance and a further improvement of diagnostic and therapeutic techniques.

Equally important is to follow the growth path of those born at risk in its psycho-neuro-evolutionary and auxological aspects by collaborating with their families; not least it is important to evaluate their quality of life at a distance from birth. In this regard, we hope that further scientific research will be carried out to make a greater contribution to the knowledge of the subject.

## Abbreviations

AGA: Appropriate for Gestational Age
ELBW: Extremely Low Birth weight
LGA: Large for Gestational Age
NIDCAP: Newborn Individualized Developmental Care and Assessment Program
SGA: Small for Gestational Age
VLBW: Very Low Birth weight
